# Author Correction: *Wolbachia cifB* induces cytoplasmic incompatibility in the malaria mosquito vector

**DOI:** 10.1038/s41564-022-01098-9

**Published:** 2022-03-07

**Authors:** Kelsey L. Adams, Daniel G. Abernathy, Bailey C. Willett, Emily K. Selland, Maurice A. Itoe, Flaminia Catteruccia

**Affiliations:** Department of Immunology and Infectious Diseases, Harvard T.H. Chan School of Public Health, Boston, MA USA

**Keywords:** Microbial genetics, Molecular biology

Correction to: *Nature Microbiology* 10.1038/s41564-021-00998-6, published online 24 November 2021.

In the version of this article initially published, there was an error in the label of the bottom-left construct in Fig. 1a. The label has been corrected to read “*vasa-cifB*.” The change has been made to the HTML and PDF versions of the article.

**Fig. 1:**
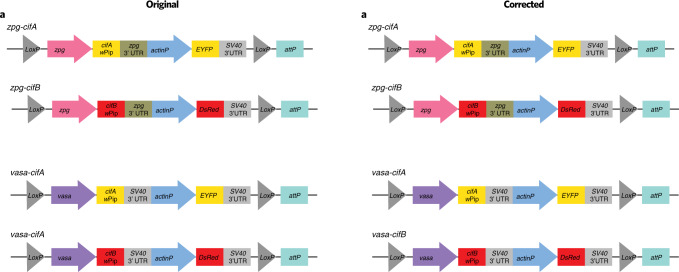
Original and Corrected.

