# Supplementary material for: Wolbachia cifB induces cytoplasmic incompatibility in the malaria mosquito vector
Source: Nat Microbiol. 2021 Nov 24;6(12):1575–82. doi: 10.1038/s41564-021-00998-6 (PMC8612931; doi:10.1038/s41564-021-00998-6)
Supplement: Supplementary file 1 — Reporting Summary. [file 41564_2021_998_MOESM1_ESM.pdf]

nature research

Corresponding author(s): Flaminia Catteruccia

Last updated by author(s): September 28th, 2021

Reporting Summary

Nature Research wishes to improve the reproducibility of the work that we publish. This form provides structure for consistency and transparency in reporting. For further information on Nature Research policies, see our Editorial Policies and the Editorial Policy Checklist.

Statistics

For all statistical analyses, confirm that the following items are present in the figure legend, table legend, main text, or Methods section.

n/a	Confirmed	
☐	☒	The exact sample size (n) for each experimental group/condition, given as a discrete number and unit of measurement
☐	☒	A statement on whether measurements were taken from distinct samples or whether the same sample was measured repeatedly
☐	☒	The statistical test(s) used AND whether they are one- or two-sided<br>*Only common tests should be described solely by name; describe more complex techniques in the Methods section.*
☐	☒	A description of all covariates tested
☐	☒	A description of any assumptions or corrections, such as tests of normality and adjustment for multiple comparisons
☐	☒	A full description of the statistical parameters including central tendency (e.g. means) or other basic estimates (e.g. regression coefficient) AND variation (e.g. standard deviation) or associated estimates of uncertainty (e.g. confidence intervals)
☐	☒	For null hypothesis testing, the test statistic (e.g. F, t, r) with confidence intervals, effect sizes, degrees of freedom and P value noted<br>*Give P values as exact values whenever suitable.*
☒	☐	For Bayesian analysis, information on the choice of priors and Markov chain Monte Carlo settings
☒	☐	For hierarchical and complex designs, identification of the appropriate level for tests and full reporting of outcomes
☒	☐	Estimates of effect sizes (e.g. Cohen's d, Pearson's r), indicating how they were calculated

Our web collection on statistics for biologists contains articles on many of the points above.

Software and code

Policy information about availability of computer code

Data collection	N/A
Data analysis	N/A

For manuscripts utilizing custom algorithms or software that are central to the research but not yet described in published literature, software must be made available to editors and reviewers. We strongly encourage code deposition in a community repository (e.g. GitHub). See the Nature Research guidelines for submitting code & software for further information.

Data

Policy information about availability of data

All manuscripts must include a data availability statement. This statement should provide the following information, where applicable:

- Accession codes, unique identifiers, or web links for publicly available datasets
- A list of figures that have associated raw data
- A description of any restrictions on data availability

Raw data from Figures 1-4 is available in an Excel file format as Source data. Codon-optimised DNA sequences are deposited in GenBank under the Accession codes OK352257 (cifA) and OK352258 (cifB).

Field-specific reporting

Please select the one below that is the best fit for your research. If you are not sure, read the appropriate sections before making your selection.

☒ Life sciences ☐ Behavioural & social sciences ☐ Ecological, evolutionary & environmental sciences

For a reference copy of the document with all sections, see nature.com/documents/nr-reporting-summary-flat.pdf

Life sciences study design

All studies must disclose on these points even when the disclosure is negative.

Sample size	We conducted power analysis in G*Power 3.1 to detect a 50% reduction in fertility yielding n=5, non-centrality parameter = 21.3, critical chi-squared result of 11.07, total samples size =10 to estimate the sample size required for detecting differences. On average we used far greater sample sizes than this power analysis suggested, as we planned to use more stringent tests for non-parametric data (which cannot be estimated by power analysis).
Data exclusions	No data were excluded.
Replication	All fertility and egg development phenotypes observed in different groups were confirmed in a minimum of two experiments to ensure reproducibility. Observations were consistent between replicates. Embryo imaging experiments were performed only once as the number of embryos obtained is representative of a large sample size. While fixing and imaging of ovaries was performed only once, observations of ovarian defects were consistent among dissected females in three replicates.
Randomization	As organisms were taken from different transgenic colonies to compare during experiments, randomization of individuals was not possible, although care was taken to standardize rearing between colonies.
Blinding	Groups were not blinded but were rotated between researchers for different replicates to reduce bias of data collection.

Reporting for specific materials, systems and methods

We require information from authors about some types of materials, experimental systems and methods used in many studies. Here, indicate whether each material, system or method listed is relevant to your study. If you are not sure if a list item applies to your research, read the appropriate section before selecting a response.

Materials & experimental systems

n/a	Involved in the study	
☒	☐	Antibodies
☒	☐	Eukaryotic cell lines
☒	☐	Palaeontology and archaeology
☐	☒	Animals and other organisms
☒	☐	Human research participants
☒	☐	Clinical data
☒	☐	Dual use research of concern

Methods

n/a	Involved in the study	
☒	☐	ChIP-seq
☒	☐	Flow cytometry
☒	☐	MRI-based neuroimaging

Animals and other organisms

Policy information about studies involving animals; ARRIVE guidelines recommended for reporting animal research

Laboratory animals	Anopheles gambiae mosquitoes were used from the G3 strain. Species was confirmed using specific primers (Santolamazza et al. 2008, Malar J.). Both males and females were used for experiments. For fertility and egg development experiments, 6-8 day-old females were blood fed and allowed to oviposit. For RNA samples, 3-6 day-old males and females were dissected.
Wild animals	There were no wild animals in this study
Field-collected samples	There were no field-collected samples in this study
Ethics oversight	Approval was received by the Committee on Microbial Safety to create transgenic mosquitoes with Wolbachia-derived genetic sequences

Note that full information on the approval of the study protocol must also be provided in the manuscript.
